# Diabetes

**Published:** 1905-06-10

**Authors:** 


### DIABETES.

(*Continued from page* 175.)

**The Pancreas and Diabetes.**—The normal pancreas contains, according to Sauerbeck,[13] from 3 to 10 islands of Langenhans in an area of 3·7 c.mm., the number varying in different parts. He notes the resistance to disease shown by the islands in chronic interstitial pancreatitis and in cancer, whereas in lipomatosis without diabetes they may be extensively damaged. In only 3 of 17 cases of diabetes was there extensive disease; in 11 there were localised areas of sclerosis, interacinar sclerosis, lipomatosis, or other moderate changes. In more than half there was definite change in the islands. No case of extensive disease of the islands has been reported without diabetes, but the reverse does not hold good. In some cases there may be perverted function of the islands, in others the liver or nervous system may be at fault. Pearce[14] states that if one-fourth of the pancreas is left *in situ*, or is transplanted elsewhere, diabetes does not occur. The form of pancreatitis responsible for diabetes is the chronic interacinous, in which the islands are involved. In 6 cases of diabetes 4 showed arterial changes involving the islands, one degeneration of those bodies, and in one there was no pancreatic lesion, but fibrous nodules in the suprarenals. Cancer acts by obstructing the pancreatic duct, and thus setting up chronic inflammatory changes, but complete destruction of the gland may occur without diabetes. In 30 cases of cancer three had glycosuria, but only one true diabetes. In health 250 gms. of sugar will cause glycosuria, in disease much smaller quantities. Nearly half the cases of diabetes show no pancreatic changes. Pearce also notes the resistance of the islands to cancer. Diabetes associated with acromegaly is due to induration of the pancreas. Edsall[15] believes that the internal secretion of the pancreas merely regulates a glycolytic ferment derived from other tissues. It has been shown that pancreatic and tissue juices are only glycolytic when combined, like the complement and immune body; they cannot act separately. Diabetes, however, is not due to imperfect sugar destruction only; the varying powers of patients to assimilate different sugars disproves this. Possibly there is deficient power to form glycogen, or very rapid conversion of that substance into sugar. Dickinson[16] holds the same view, believing that more sugar is produced than can be dealt with by normal tissues. Lancereaux[17] points out that microscopic examination of the islands is always necessary to determine the existence of a pancreatic diabetes, which he regards as identical with "wasting" diabetes. He notes that in 167 collected cases the islands were diseased in 130, and records four cases of typical diabetes in which the changes in the islands were well marked. Finney[18] points out that there may be in diabetes no lesions in the islands or only slight ones; in these cases a functional change may be present. The number of the islands may be variously decreased, or even reduced to *nil*. No extensive changes occur in them without diabetes, and the lesions may be a primary hyaline degeneration.

**The Liver and Diabetes.**—Engorgement and exudation are the principal changes met with in the liver, according to Dickinson.[19] He attributes these to alterations in vascular tone, which he thinks underlie the diabetic condition in some cases. Pavy and Siau[20] question Bock and Hoffmann's statement that shutting off the liver from the circulation causes a disappearance of sugar within three-quarters of an hour. Even after some hours they find 5 per cent. of sugar present, but there are great variations in different experiments, and at different times in the same experiment.

**The Thyroids, Suprarenals, and Diabetes.**—It has been pointed out by Lorand[21] that there are etiological and symptomatic relationships between diabetes, Graves' disease, and acromegaly. When the thyroid is extirpated, hypertrophy of the pituitary body may occur, as in myxœdema and cretinism; when the pituitary body is removed, hyperthyroidism and glycosuria may result. The alimentary glycosuria of Graves' disease may be due to over-activity of the thyroid, together with alterations in the islands of Langerhans, and there is probably some antagonism between the pancreatic secretions and those of the thyroid and suprarenals. Hence in Addison's disease glycosuria is rare, when the thyroid secretion is defective; but common in Graves' disease, when it is excessive. In lactation, pregnancy, and thyroid feeding, glycosuria may also occur, and also in acromegaly when there are symptoms of increased thyroid action. In diabetes the thyroids are often slightly enlarged. He considers that overactivity of the thyroid causes degeneration of the pancreas and *vice versâ*; the internal secretion of the islands of Langerhans neutralises toxins formed in the thyroid, hence bad diabetes occurs in young persons when the thyroid is active, and slighter cases in old persons. Temporary glycosuria occurs when the pancreas is normal, and only the thyroid secretion excessive. Lorand has obtained remarkable results in 10 cases of diabetes by means of serum from an animal in which the thyroid had been removed. Parker [22] has recorded a case of "Bronzed diabetes" in which post-mortem there was monolobular cirrhosis of the liver with numerous pigment granules, fibrosis and pigmentation of the spleen and pancreas, pigmentation of the suprarenals and some atheroma of the aorta. He considers the condition due, as Crofton suggested, to deficiency of trypsin; the sugar is thus not destroyed, and the hæmoglobin instead of being normally changed by trypsin is converted into hæmochromogen. The cirrhotic condition of the liver causes this pigment to accumulate, and adds the symptom of bronzing to that of glycosuria. Beattie,[23] who records a case with very similar appearances, ascribes the pigmentation to the action of a toxin derived from the intestine which causes cirrhosis and consequent inability of the cells to properly deal with the pigments present. The diabetes he regards as a late manifestation of the disease, though in his case it appeared before pigmentation was observed. Pearce [24] attributes bronzed diabetes to coincident cirrhosis of the liver and pancreas.

**Diabetes and Albuminuria.**—Statistics showing the proportion of diabetic cases presenting albuminuria are shown by Vas [25] to vary greatly, probably owing to different methods of examination and different interpretations of the results. The highest proportion was 79·4 per cent., and the lowest 23·5 per cent. Vas divides the cases into two main classes: one in which the albuminuria is due to some cause, such as gout, cystitis, etc., which has nothing to do with the diabetes, and one in which it is an integral part of the disease. These again are subdivided into three classes:—(1) Where there is no evidence of renal changes clinically or morphologically. These are the most numerous; the amount of albumen is slight and transient, and, though generally regarded as functional, post-mortem there is often evidence of renal change. (2) Where there is morphological evidence of kidney change in the shape of granular or hyaline casts, but no other renal symptoms. The albumen is often present in large quantities and often varies indirectly with the amount of sugar excreted. (3) Where there are also clinical signs of kidney disease. In these the polyuria has been thought to cause an interstitial nephritis, and often renal symptoms predominate in the end, the sugar disappearing from the urine. This may be due to the damaged renal cells being unable to excrete it. The prognosis is always bad. Hirschfeld [26] states that nephritis occurred in 11 per cent. of his cases, and that all diabetics suffer to some extent from cystitis. He thinks the nephritis due to kidney irritation, and not to arteriosclerosis as Senator suggests. Kraus [27] has shown by experiments on kittens artificially rendered glycosuric, that even when all the reserve of glycogen is excreted, sugar continues to be formed from the tissues.

**Acetonuria.**—The effect of fats in the production of acetonuria is illustrated by a case reported by Pollatschek.[28] The patient had suffered from diabetes for years, with acetone, diacetic acid, and $\beta$-oxybutyric acid in the urine. He was treated with a very fatty diet, and whilst under this system developed bronchitis with some symptoms of coma. A year later, the fats were gradually eliminated from his diet, especially butter, with the result that the acetone disappeared almost entirely, his general condition remaining good. Joslin [29] criticises the experimental work on this subject, owing to the facts that in many the diacetic acid and $\beta$-oxybutyric acid are not taken into account, the pulmonary excretion of acetone is not measured, the effect of glycerin in neutral fats (which inhibits acetone formation) is not allowed for, and the absorption of fat is not measured. Admitting that not only butter, but fats poor in volatile fatty acids increase acetonuria, and that carbohydrates diminish it, Joslin has shown that neutral fats, either higher or lower in the series, do not increase acetonuria on account of their glycerin component. Oleic acid produces marked acetonuria, but not butyric acid. Palmitic and stearic acids are so little absorbed as to produce no effect. Beauvy [30] points out that acetone occurs in the urine under a variety of conditions, and as a rule only gives rise to symptoms when present to the extent of ·2 gm. per litre. It is increased by meat diet and fats and diminished by carbohydrates. Its origin is thought by some to be derived from sugar by the action of alcohol or aldehyde, by others from albuminoids. All experimental diets for producing acetonuria are really starvation diets, and hence theories based on them are not conclusive. Acetone is slightly toxic to healthy persons, and much more so to those with hepatic disease or diabetes. It passes readily into the urine in these cases as if the liver could not arrest it.

[13] Amer. Jour. Med. Sci., March 1905, p. 559. [14] Edin. Med. Surg. Jour., Nov. 1904, p. 447. [15] Ibid., p. 450. [16] Loc. cit. [17] Birm. Med. Rev., Sept. 1904, p. 511. [18] Ibid., p. 512. [19] Loc. cit. [20] Birm. Med. Rev., Sept. 1904, p. 509. [21] Edin. Med. Surg. Journ., Nov. 1904, p. 448. [22] Birm. Med. Rev., Sept. 1904, p. 513. [23] Ibid., p. 515. [24] Loc. cit. [25] Amer. Jour. Med. Sci., Feb. 1905, p. 350. [26] Med. Rec., Dec. 24, 1904, p. 1026. [27] Birm. Med. Rev., Sept. 1904, p. 513. [28] Brit. Med. Jour. Epit., 1904, No. 370. [29] Med. Rec., Nov. 26, 1904, p. 859. [30] Med. Rec., Dec. 24, 1904, p. 1031.

